# Supplementary material for: Recommending encounters according to the sociodemographic characteristics of patient strata can reduce risks from type 2 diabetes
Source: PLoS One. 2021 Apr 12;16(4):e0249084. doi: 10.1371/journal.pone.0249084 (PMC8041209; doi:10.1371/journal.pone.0249084)
Supplement: S1 Appendix — (DOCX) [file pone.0249084.s001.docx]

**Appendix**

**Estimates for Positive Count Model of Physical and Telephonic Encounters**

**Table comparing the clustering by K-means and LCA methods.**

s
