# Supplementary material for: Recommending encounters according to the sociodemographic characteristics of patient strata can reduce risks from type 2 diabetes
Source: PLoS One. 2021 Apr 12;16(4):e0249084. doi: 10.1371/journal.pone.0249084 (PMC8041209; doi:10.1371/journal.pone.0249084)
Supplement: Attachment — Submitted filename: Response to Reviewers.docx [file pone.0249084.s002.docx]

**Point-by-point Responses to the Review Team’s Comments**

*(All excerpts from the revised paper are in quotes and italics)*

The authors would like to thank the academic editor and the reviewers for the careful reviews and the constructive comments they provided to improve the quality of the manuscript. We have revised our manuscript based on the review feedback. The following are point-by-point responses to address all reviewers’ comments.

**Reviewer #1**: My thanks to the editor and authors for the opportunity to review this manuscript. This manuscript reports the results of a retrospective study of the effect of social and demographic variables on blood glucose among persons with diabetes. It is an important topic and this manuscript fills an important gap in the research literature. Below I provide some comments and suggestions to the editor and authors that also serve as rationale for my recommendation. Overall, I do not think the manuscript is appropriate for publication but the authors might consider a separate and new analysis taking into account some of these critiques.

**Response**. The authors would like to thank you for your time and effort in reviewing the manuscript and providing us with constructive and thoughtful comments. We appreciate that you found the topic ‘important’ and that the paper ‘fills an important gap’. We have tried to address all your concerns meticulously in the revised manuscript. Your comments have helped us improve the paper by making the analysis more robust and improving the exposition and positioning. Below we respond to each of your comments and feedback in detail.

Minor: I do not see what “customizing” has to do with the hypothesis being tested in this manuscript. This study does not seem to be about customization, but rather recommendations for care utilization patterns.

**Response**. We agree with your comment, and have revised the writing accordingly. Specifically, we have changed the title to “Recommending Encounters According to the Sociodemographic Characteristics of Patient Strata Can Reduce Risks from Type 2 Diabetes” to reflect this change in positioning. In addition, we have changed the text of the paper to address this point.

Abstract:
• The abstract begins with a description of the importance of physician encounters and “the inverse law of patient encounters” which is seems to be mostly conventional wisdom rather than established theory. I recommend removing the first two sentences and providing a short and clear description of the rationale for the paper, grounded in literature on socioeconomic status and health care utilization. A more straightforward statement of the primary hypothesis would be clearer than the current presentation in the abstract.

**Response**. We have removed the sentence about “inverse law of patient encounters”. We have now re-written the abstract based on your comment and have made the abstract more direct. Specifically, the first paragraph of the abstract directly states the primary objective of the paper. For your reference the excerpt from the revised manuscript is provided below.

*“Objectives: Physician encounters with patients with type 2 diabetes act as motivation for self-management and lifestyle adjustments that are indispensable for diabetes treatment. We elucidate the sociodemographic sources of variation in encounter usage and the impact of encounter usage on glucose control, which can be used to recommend encounter usage for different sociodemographic strata of patients to reduce risks from Type 2 diabetes.”*

• The abstract includes unnecessary stylistic language (e.g. “last but not least”) that detracts from the message.

**Response**. Based on this comment and the previous one, we have changed the language significantly to make the abstract precise and direct.

• Description of “do correlate” should be accompanied by correlation coefficient or other statistic that illustrates this finding.

**Response**. Thanks for this great input. We have deleted that sentence, and now directly state the results. Excerpt from the revised manuscript is provided below.

*“****Results and Conclusions:*** *Conditional on patients’ clinical status, demographics, and insurance status, significant inequality in patient encounters exists across ZIP codes with varying sociodemographic characteristics. One additional physical encounter in a six-month period marginally increases the probability of transition from a diabetic state to a pre-diabetic state by 4.3% and from pre-diabetic to the non-diabetic state by 3.2%. Combined marginal effect analyses illustrate that a ZIP code in the lower quartile of high school graduate percentage among all ZIP codes has 1 fewer physical encounter per six months marginally compared to a ZIP code at the upper quartile, which gives 5.4% average increase in the probability of transitioning from pre-diabetic to diabetic. Our results suggest that policymakers can target particular patient groups who may have inadequate encounters to engage in diabetes care, based on their immediate environmental sociodemographic characteristics, and design programs to increase their encounters to achieve better care outcomes”*

• The abstract seems to confuse segregation (measured as African Americans in a zip code) with socioeconomic status. The authors need to be accurate in their description of concepts.

**Response**. We agree that race is a demographic factor rather than a socioeconomic factor. We have corrected this by replacing “socioeconomic” with the terminology “sociodemographic” which includes income, education, gender, race, etc. We have corrected the entire manuscript based on this comment.

Manuscript
• The body of the manuscript has some grammar problems. I have only mentioned the first few to appear. The authors should edit and revise their work more carefully. For example:

**Response**. In this round of revision, we have carefully edited the language to improve the readability and correct the grammar. We have corrected all the following issues.

o Pg 3 ln. 60 is improperly worded. The prevalence did not increase with age, the prevalence is higher among older persons.

**Response**. We have corrected this error, thanks for pointing out. The revised sentence is provided below.

*“The incidence rate of diabetes increased with age, and reached 25.2% among those aged 65 or older [1].”*

o Pg. 3 ln 62 “in 2017 is $327 billion” should be “was”

**Response**. The error was corrected.

• Pg 5 ln 103 “glucose transitions” is not defined or referenced until much later in the paper and most readers will not understand what is being referred to here. The transition approach is elaborate and does not appear to have been reported or described elsewhere. The authors likely need to establish the validity of this approach in a separate manuscript.

**Response**. Thanks for pointing these out. We added one paragraph in the Introduction section to explain how glucose transitions are defined (lines 87-98), please see the excerpt below.

*“According to American Diabetes Association, blood sugar levels can be measured by Hemoglobin A1c (A1C), Fasting Plasma Glucose (FPG), Oral Glucose Tolerance Test (OGTT), etc. The current medical diagnosis standard classifies a patient glucose level into three states: Diabetic (A1C ≥ 6.5%; FPG≥126 mg/dl; OGTT≥200 mg/dl), Pre-diabetic (5.7%≤A1C < 6.5%; 100mg/dl≤FPG<126 mg/dl; 140 mg/dl≤OGTT<200 mg/dl), and Normal (A1C<5.7%; FPG<100 mg/dl; OGTT<140 mg/dl). The glucose level of a patient with type 2 diabetes may fall in any of the three states at a time point, depending on various factors such as self-management and efficacy of treatments. The change in patients’ glucose value over a time period is often used as a measure of glucose control [18–20]. The transition among the three states of glucose over time periods of a patient reflects their disease progress and the effectiveness of disease management. For instance, a transition from Diabetic to Normal in a six-month time period indicates effective disease control, while a transition from Normal to Diabetic indicates disease worsening.”*

In order to support the transition approach, we added discussion in the Study Data and Methods section (lines 262-273) about why we used glucose state transition (as opposed to the change in glucose value) as the dependent variable, and why the multinomial regression model is used for modeling transitions. Please see the excerpt below.

*“The transition probabilities depict how the health risk changes may depend on a patient’s engagement via physical and telephonic encounters, as well as the patient’s sociodemographic characteristics, which are partially characterized by the ZIP-code level variables. We then build a multinomial logistic regression model [28] to estimate the effect of encounters on glucose status transitions, including ZIP-code level sociodemographic variables and their interactions with the encounter variables. Multinomial logistic regression is a well-developed tool for modeling transitions among a finite number of states, and has been widely adopted in various scientific fields [29–31]. Using the transition of glycemic state as a dependent variable has the advantage of model flexibility such that the effect of an explanatory variable is allowed to vary by different transitions. In contrast, directly using the change in glucose value as a response variable assumes unvaried effect of an explanatory variable, regardless of a patient’s current glycemic state. The multinomial logistic regression model for glucose state transitions is stated as follows.”*

• Pg 5 which IRB approved the study?

**Response**. The research has been approved as **exempt** for IRB review by the Office for the Protection of Research Subjects at the University of Illinois Urbana-Champaign.

• Pg 5 How were the data collected? Chart abstraction? Electronic medical record? What procedures were used?

**Response**. The data was collected from the Electronic Medical Records of the study clinic. The clinic’s IT team extracted the variables from the electronic medical records of patients who visited the clinic for diabetes treatment. We have clarified the data source in the paper.

• Given that patients have A1C values, why use FPG as the outcome instead of A1C?

**Response**. We have used the FPG measure because FPG measures are more frequently available, whereas the A1C measures are less frequent for the patients. Since the A1C measures are not consistently available for all patients, we are using the FPG measures.

• Were A1C and FPG point of care or from a venous blood draw in the lab?

**Response**. The measures are from lab tests with venous blood draw. All clinical lab measures are from the lab database of the clinic.

• Zip codes are large and potentially immensely heterogeneous. Why not geocode to the census tract or block group to get more fine grained information? Tools like tidycensus and sociome provide the opportunity for higher resolution, neighborhood level analyses that do not suffer from the same degree of weakness as zip codes.

**Response**. We agree that finer geocode level analysis will be much better. However, due to confidentiality issues the clinic has made the zip codes of patients available, but the neighborhood information was not provided. This is because many of the zip codes are rural zip codes which are sparsely populated, and any finer than zip code level information can potentially lead to the identification of the patients with gender, age, and other demographic information.

• Major: The manuscript is presented as though all patients had complete data, but in my experience that is rarely the case. How many patients were excluded due to incomplete data?

**Response**. As you rightly mentioned, all patients did not have data over the entire period. We have now described the data organization in more detail. Please see the excerpt from the revised manuscript about data inclusion/exclusion.

*“The data, collected at a multi-facility clinic in central Illinois, ranges from 02/01/2013 to 12/21/2015 and includes clinical and demographic information of 10,235 patients with at least one indication of high blood glucose, whose identifying information has been removed before the study. In the data set, there are two metrics that measure patients’ glycemic levels: A1C and FPG. All the patients in the sample had their FPG measure as well as A1C measure. However, FPG measures were taken on patients at regular intervals and the A1C measures, which are more robust, were taken while identifying the diabetes condition at a less frequent interval. Therefore, we used the A1C measures along with ICD-10 diagnosis codes to identify diabetes patients, and used the FPG measure to model the diabetes status of patients. We used the ICD-10 codes to select the final sample. The primary disease codes for all types of diabetes are E08, E09, E10, E11, O24, and E13. However, we removed all patients whose disease codes are E08, E09, or E10, which indicate diabetes due to other underlying conditions (23.3% of patients), drug-induced diabetes (13.1% of patients), and Type I diabetes (4.4% of patients). Furthermore, we removed all ICD codes O24 (pre-natal diabetes) and E13 (miscellaneous condition related diabetes), totaling 8.8% of the patients. Patients with Type II diabetes due to other underlying conditions are not usually chronic, and the diabetes usually subsides after the underlying condition is cured. Furthermore, we are interested in observing the effect of continued and regular encounters, both physical and telephonic, on diabetes outcome for the population of patients. Therefore, we removed all patients who do not have sufficient records in the dataset. For example, many patients joined the system only towards the end of the observation period and do not have sufficient records to warrant analysis. In addition, some patients dropped out in the middle of the observation period due to deaths, mobility, or other personal causes. We retained all patients who had records for more than 80% of the observation timeframe. A total of 29.6% of the data was removed due to the non-availability of data. A total of 2124 patients (20.8% of total initial record) were retained in the final sample of patients. We checked that patients removed from the data due to unavailability of sufficient observations do not have any systematic gender or zip code based variation. These 20.8% of the patients corresponds to 46.3% of the total number of encounters. The time range of the study is divided into 6-month time periods. For each patient at each time period, the data set includes the following variables: glucose (mg/dl), LDL cholesterol (mg/dl), age (years), gender, number of physical encounters, number of telephonic encounters, health insurance policy, ZIP-code of patient home.”*

• Major: The methods section does not appear to describe inclusion/exclusion criteria.

**Response**. We have now described the data organization process in a step by step manner including the inclusion and exclusion criterion. Please see the excerpt provided in the previous response.

• What are the tests for which p values are reported in Table 1? Are there hypotheses tied to these tests?

**Response**. We have now described the types of the tests and the associated hypotheses of the p-values in Table 1 (the bottom row).

• Table 1 refers to “clusters” but insufficient explanation in the table is given about what is meant by “cluster” How did the authors decide to create these clusters?

**Response**. We have now added a subsection called Exploratory Analysis (lines 165-205). In this section, we first discuss the motivation of the clustering process and the rationale more succinctly, before Table 1 is presented. The clustering results in Figure 1 show that significant variation exists in patient encounter usage across different sociodemographic clusters within the same clinical cluster. These results clearly indicate potential inequalities in patient encounters across ZIP-codes with varying sociodemographic characteristics, after controlling for patients’ clinical status. The clusters motivate us to build Model 1 to analyze the effects of zip-code level sociodemographic characteristics on encounter usage. In addition, the clustering results can further help analyze encounter usage within a clinical cluster in which patients have similar clinical measures.

• The state transition framework for office measurements of FPG is somewhat innovative and potentially useful.

**Response.** Thank you for your appreciation, we have now made the state transition model a more integral part of the paper.

• Too little information is provided about the number and frequency of measurements of FPG per patient.

**Response.** In the final data there are 12533 FPG measures, with an average frequency of 5.901 (std. dev: 1.3) per patient. This means all patients have one measure on average in a six month period with some variation.

• It seems that patients with insufficient follow-up visits would be excluded because they would have no transition data. This is a potential limitation of the design. Doesn’t this mean that Model 2 should come first? Wouldn’t the probability of having repeated visits influence the frequency of measurement and thus the potential to observe a glucose transition?

**Response**. We agree that some of the patients have infrequent FPG measures, and therefore have insufficient data on transition. However, we submit that the sample size is sufficient to build and justify our statistical models. Thank you for suggesting that Model 2 should come first to make the flow of analysis more logical and smoother. We have revised the manuscript and changed the sequence of models. The sequence of analyses is as follows.

1. We describe the descriptive statistics and the clustering analysis, which motivates the model that comes next.
2. We show that encounter usage is associated with zip-code level sociodemographic explanatory variables (Model 2 in the previous version).
3. We estimate the effect of encounter usage on diabetes state transitions (Model 1 in the previous version).

• IT does not seem to make sense that the cluster analysis is embedded in model 2. Should not the cluster analysis be presented as having its own analytic plan and hypotheses? What was the rationale for this step other than data reduction? How do these locally, empirically defined clusters compare to other approaches of transforming zip code and clinical measurement data?

**Response.** Thank you for making these observations that have led us organize the paper for better flow. We have revised the manuscript such that the cluster analysis is no longer embedded in model 2. Instead, the clustering analysis has its own section (lines 165-205). The purpose of the cluster analysis has been now described more clearly. First, the clustering results show that encounter usage is heterogeneous across different sociodemographic clusters, within the same clinical cluster. This phenomenon motivates us to build the model to explain encounter usage more explicitly. Second, the clinical clusters are used as controls in the model, in order to achieve finer tuned estimates within each clinical cluster. While we have not included hypotheses related to the clusters, we have now explained the cluster analysis more clearly.

• K-means cluster analysis is notorious for having problems with reproducibility. This is a serious limitation of the analysis. The authors should recognize this limitation and consider or propose future working using more sophisticated techniques like latent class analysis or factor mixture models.

**Response**. We agree that K-means clustering has a random element in it. Therefore, the usual procedure is to have multiple random starts for robustness. We have done 25 random starts for K-means clustering, and the clustering results are very similar. We have recognized this as a limitation in the revised manuscript. We also tried latent class analysis (LCA) as you suggested. In the Appendix, we provide a table comparing the clustering by K-means and by LCA. We find that the differences between the patient clusters are insignificant. Please see below the excerpt of the added paragraph regarding K-means.

*“The clustering was done with K-means clustering. The advantage of K-means clustering is that it does not require distributional assumptions, and can be done in a non-parametric setting. However, K-means depends on the initial starting points, and therefore tends to suffer from lack of stability in some cases [27]. Therefore, we performed the K-means with 25 random starts and used the most frequent clustering. For the study data, all random starts provided similar clustering results with less than 1% error on average. Finally, we re-estimated the K-means clustering multiple times to ensure stability. Furthermore, in the Appendix we provide a table comparing the clustering by K-means and by Latent Class Analysis (LCA). We find that the differences between the patient clusters are insignificant.”*

• Major weakness: Detailed results of the cluster analysis are largely omitted from the manuscript.

**Response**. We have provided an appendix that provides the detailed analysis of the cluster analysis.

• There are many comparisons made in Table 2.
• Table 2 should have a sample size. No mention is made of whether there was any adjustment for multiple comparison, drawing into question the validity of any of the bolded “significant” findings. The authors need to address for multiple comparison in this type of table, or if they have done so, should say that they did. Significance tests are a poor and possibly invalid approach here anyhow (see American Statistical Association recent public statements on this matter) and 95% confidence intervals for each estimate would be much preferred.

**Response**. As suggested, we have provided 95% confidence intervals for the results.

• Table 2 seems a little bit more like an all by all table of raw output rather than a presentation of results. With so many interaction effects the results presentation is both bewildering and suspect.

**Response**. We have revised the presentation of the table to make it look more organized. We have also significantly revised the manuscript to make sure that the table are carefully interpreted by the text. Please see the subsection: Effect of Encounters on Glucose Transition (lines 584-679) for the interpretation of the table.

• All of the interpretations about results surrounding the larger percentage of African Americans in a zip code are suspect. This is especially concerning given that “Cluster 2” has just 8% African American population. Are we to believe that there is something special about zip codes with 8% vs. 4% African Americans as it relates to diabetes care? What theory in the literature would suggest this is important for us to look at?

**Response**. In the previous version, we used the zip-code variable percentage of African American as an illustration on how the two models can be combined together to derive the marginal effect of sociodemographic factors on encounter usage, and further on glucose state transitions. We deem that all sociodemographic factors discussed in the manuscript (income, education, and race) are important explanatory variables. We have revised the manuscript, especially the interpretation of results, to make sure that (i) the effects of all the concerned sociodemographic variables are carefully interpreted, and (ii) make it clear that the variables discussed in the marginal analysis are for the purpose of illustrating the effect size from the two models, and that we are not limiting our study on that particular variable. Indeed, there exists a large body of literature on the association between race and healthcare outcomes and access (Cheng et al. 2019; Choi et al. 2016; Gaskin et al. 2014; Sambamoorthi and McAlpine 2003; Williams et al. 2016). We have now provided literature support for the different sociodemographic factors considered. Please see lines 147-158 in the revised manuscript.

• Although there are good reasons to examine socioeconomic status and glucose control, and the authors appear to have assembled a useful regional data resource, the analytic plan is insufficiently described and poorly justified. Critical details are omitted from the manuscript (missing data, inclusion/exclusion criteria). The analytic methods and reported results, especially the clustering, the multiple comparisons in Table 2 and the many inferences made about small observed magnitude differences render the results and discussion unable to convince this reviewer of the accuracy, relevance or salience of any of the study findings.

**Response**. We have tried to address all your comments and feedback. Hopefully, you will find a much improved paper, and we are able to convince you of the value of the study. We sincerely acknowledge the value of your comments and feedback in improving the paper. Your thoughtful comments have been extremely useful and instrumental in improving the paper.

**Reviewer #2**: 1. The study design was clearly laid out for the reader. Variables of interest were well defined for the reader.
2. The statistical methods were clearly explained in the paper and appropriate to the research question.
3. The statistical methods were appropriate to analyze the variables of interest and the rationale for the researcher(s) choice(s) of statistical methods was explained.
4. The subject matter was relevant to diabetes care and to more general patient care areas.
5. The subject matter of the study is an important area of concern regarding diabetes care outcomes in varying sociodemographic settings.
6. Allocation of medical care is based on a number of variables. The variables of interest in the study are some of the variables identified in other previous studies that examine the role of social determinants of health in population care outcomes.
7. This study adds to the body of knowledge in the subject area of diabetes care outcomes by identifying another aspect of care models and delivery of health care services to address areas of greater need.
8. Contentions of the author(s) were supported by the data.

The article was a very technical read in terms of data analyses and discussion of results. It contributes further to the body of knowledge in the subject area.

**Response**. We sincerely acknowledge our gratitude for your appreciation. The author team is thankful for your time and effort in reviewing the paper, and your positive and encouraging comments. We are happy that you have found the paper satisfactory and valuable.

**Reference**

Cheng, Y. J., Kanaya, A. M., Araneta, M. R. G., Saydah, S. H., Kahn, H. S., Gregg, E. W., Fujimoto, W. Y., and Imperatore, G. (2019), “Prevalence of Diabetes by Race and Ethnicity in the United States, 2011-2016,” *JAMA - Journal of the American Medical Association*, American Medical Association, 322, 2389–2398. https://doi.org/10.1001/jama.2019.19365.

Choi, S. H., Terrell, J. E., Fowler, K. E., McLean, S. A., Ghanem, T., Wolf, G. T., Bradford, C. R., Taylor, J., and Duffy, S. A. (2016), “Socioeconomic and Other Demographic Disparities Predicting Survival among Head and Neck Cancer Patients,” *PLOS ONE*, (S. M. Langevin, ed.), Public Library of Science, 11, e0149886. https://doi.org/10.1371/journal.pone.0149886.

Gaskin, D. J., Thorpe, R. J., McGinty, E. E., Bower, K., Rohde, C., Young, J. H., LaVeist, T. A., and Dubay, L. (2014), “Disparities in Diabetes: The Nexus of Race, Poverty, and Place,” *American Journal of Public Health*, 104, 2147–2155. https://doi.org/10.2105/AJPH.2013.301420.

Sambamoorthi, U., and McAlpine, D. D. (2003), “Racial, ethnic, socioeconomic, and access disparities in the use of preventive services among women,” *Preventive Medicine*, Academic Press Inc., 37, 475–484. https://doi.org/10.1016/S0091-7435(03)00172-5.

Williams, D. R., Priest, N., and Anderson, N. B. (2016), “Understanding associations among race, socioeconomic status, and health: Patterns and prospects.,” *Health Psychology*, 35, 407–411. https://doi.org/10.1037/hea0000242.
